# Brain lesion MRI and co-related MRS spectroscopy dataset

**DOI:** 10.1016/j.dib.2025.111288

**Published:** 2025-01-11

**Authors:** Sura Riyadh Saleh, Suhad A. Yousif, Israa Qader Ahmed

**Affiliations:** a Informatics Institute for Postgraduate Studies, Iraqi Commission for Computers and Informatics, Baghdad, Iraq; b Department of Computer Science, College of Science, Al-Nahrain University, Baghdad, Iraq; c Department of Neuroradiology, Neurosurgical Hospital, Baghdad, Iraq

**Keywords:** Brain tumour, MRS, T2-FLAIR, T1 and post-contrast T1

## Abstract

Analysing brain lesions of various aetiologies necessitates imaging data with subsequent diagnostic techniques that may enable concomitant visualization of spatial anatomical entities, and aberrant molecular behaviour. To fulfil this necessity, several image types and modalities should be collected. This dataset includes MRI (Magnetic Resonance Imaging) with three modalities T2-FLAIR, T1-weighted pre- and post-contrast along with Magnetic Resonance Spectroscopy (MRS) scans for 55 patients. All patients were diagnosed with neoplastic and non-neoplastic brain lesions. T2-FLAIR and contrast-enhanced T1 MRI modalities reveal structural differences in lesions, whereas MRS affords metabolite information. In addition to the imaging data, patient metadata such as age, gender, and expert diagnosis- classify brain lesions into two types: neoplastic (low or high grade) and non-neoplastic. The data were collected between 2023 and 2024 at Al-Andalus Oncology Centre in Baghdad, Iraq, and are publicly available. This dataset is essential as it combines conventional (MRI) and metabolic (MRS) imaging with expert diagnosis information. This dataset is significant as it includes MRI and MRS images along with expert diagnoses information, offering high reuse potential in medical imaging and diagnostic research.

Specifications Table

**Table:** 

Subject	Computer Science: Computer Vision and Pattern Recognition
Specific subject area	Brain lesion detection and classification
Type of data	Table, raw NIfTI Image
Data collection	The MRI, MRS, and patient metadata, which includes general patient information, were acquired on 1.5 Tesla collected at Al-Andalus Oncology Centre. The patient's meta information is listed in the supplementary table.
Data source location	Al-Andalus Oncology Centre in Baghdad, Iraq
Data accessibility	Repository name: Mendeley Data Direct URL to data: https://data.mendeley.com/datasets/v3gwhkyjsg/3
Related research article	None

## 1 Value of the Data

• To our knowledge, this is the first publicly available dataset to include both MRI (T2-FLAIR, pre- and post-contrast T1) and MRS data to assist in classifying brain lesions.
• It also includes expert-diagnosed information that can be used to classify gliomatous lesions as low-grade or high-grade and differentiate between a tumor and a tumor-like lesion.
• Due to the rich patient information available and identification information of age, gender, and diagnosis, the data set can also be employed for demographic analysis of the presentation and progression of brain lesions community.
• It includes expert diagnosis, allowing researchers to study and develop Artificial intelligence models for classifying brain tumors as benign (low grade) or malignant (high grade) and distinguishing them from tumor-like lesions.
• Researchers can utilize the dataset to investigate the relationship between conventional MRI features and metabolic information from MRS, which may improve diagnostic accuracy.
• Given the detailed patient metadata (age, gender, diagnosis), the dataset can also study demographic trends in brain lesion differentiation and progression.

## 2 Background

The MRI is commonly used to obtain detailed brain anatomical images, especially in diagnosing brain tumors. However, mostly MRI alone cannot reaching final decision in distinguishing between brain tumors and tumor-like lesions. To address this, both MRS and MRI provide biochemical information on tissue metabolism, which helps in better diagnosis [1].

Compiling this dataset motivated us to create a comprehensive resource combining structural (conventional) and metabolic data for classifying brain lesions. By including MRI (T2-FLAIR, pre- and post-contrast T1) images and MRS data, along with expert diagnoses, this dataset helps other studies study and analyze the relationship between tissue structure and metabolic changes for better brain lesion diagnosis. By combining both MRI and MRS images, this dataset provides a significant resource [2] for medical imaging applications.

**Fig. 1:**
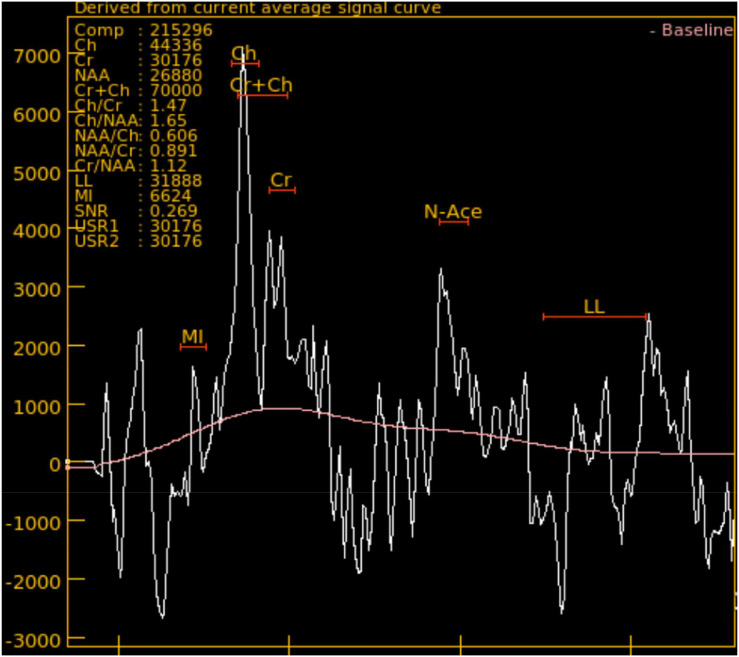
MRS spectrum for a female patient, aged 38, diagnosed with a high-grade lesion.

MRS, as shown in Fig. 1, is a type of imaging that provides metabolic information about brain tissues, used for brain diagnosis . In the contrast of MRI, MRS does not focus on structural imaging, MRS measures chemical differences to reveal concentrations of various metabolites [2,3]. MRS includes several peaks such as N-Acetylaspartate (NAA), Choline (Cho), Creatine (Cr), and Lactate (Lac), each peak have a significant key point in the diagnosing of brain lesion. Through the analysis of these peaks, MRS helps in differentiating between tumour types, evaluating the aggressiveness of the tumour and help in patient treatment future plan [4].

Furthermore, pre- and post-contrast T1-weighted MRI imaging is considered an important resource for evaluating and diagnosing brain tissue, as it gives us information related to both structural and vascular specification of brain tissue. Pre-contrast T1 imaging provides essential information to identify areas with high or low intensity, which helps in identifying brain tumor types. On the contrary, post-contrast T1 imaging is used for vascularity enhancement or blood-brain barrier rapture, which can be one of the significant features of malignant tumors [4]. In addition, it is useful for delineating the tumor margins to find a smaller lesion and active tumors. Hence, this data is very beneficial for brain tumor diagnosis research as it comprises MRI (T2-FLAIR, pre- and post-contrast T1-weighted) and MRS data in conjunction with the diagnostic information [5,6]. Using this dataset, the researcher may explore some important MRI and MRS features from each tumor that would aid in making a significant contribution toward differentiating tumor types and grades [6]. Utilizing such a database allows us to train and test models for a more accurate analysis of brain tumors that may improve the diagnosis and treatment of brain lesions.

## 3 Data Description

The dataset consists of MRI and MRS scans, along with patient metadata, structured as follows:

### 3.1 MRI Images (NIfTI format)

The MRI images were acquired using the Fluid Attenuated Inversion Recovery (FLAIR), and pre- and post-contrast for T1- weighted are considered significant MRI sequences in brain lesion diagnosis and evaluation. These images provide high-contrast structural brain information, highlighting areas affected by tumors or tumor-like lesions.

### 3.2 MRS images (NIfTI format)

MRS is considered a specialized type of imaging that has essential metabolic data about the brain tissue, especially for brain tumours. MRS makes it possible to evaluate particular metabolites in tissues of the brain and, thus, describe the microenvironment of the tumour [7]. Key metabolites analysed include:

1-Choline (Cho), a precursor of acetylcholine and a cell membrane component, and it resonates at 3.2ppm chemical shift. High amounts of Choline are a marker of cellular membrane turnover and fast cell division, therefore elevated in neoplastic lesions, demyelination, inflammation and gliosis.

2-N-Acetylaspartate (NAA) is an essential compound that assists with MRS, resonates at a 2.0ppm chemical shift, and is a marker of neuronal viability. It is, therefore, reduced in any process that destroys neurons, such as tumours, radio necrosis, and non-neuronal tumours (cerebral metastases and primary CNS lymphoma).

3-Lactate: Lactate is one of the more essential compounds that assist with MRS and resonates at 1.3 ppm chemical shift. It is a marker of anaerobic metabolism and is therefore elevated in necrotic areas (high-grade tumours) and infections (cerebral abscess).

4-Creatine (Cr): is one of the compounds examined in MRS. It resonates at a 3.0 ppm chemical shift (with a second usually smaller peak at 3.95 ppm). It is found in metabolically active tissues as the brain tends to be maintained relatively constant and is predominantly used as a convenient standard (allowing for calculating ratio).

Additionally, it is further shown that MRS may be useful in distinguishing low—and high-grade tumours based on the identified metabolite levels. For example, a high-grade glioma is characterized by high Choline, low NAA, and elevated lactate levels, meaning high cellularity and neuronal loss, but low-grade gliomas might elicit slightly different choline and lactate levels [8].

The MRS scans contain metabolic data, offering insight into the biochemical composition of brain tissues. This data is essential for distinguishing between different types of brain lesions based on their metabolic profiles [9].

### 3.3 Patient Metadata (Excel file)

Age

Gender

Expert diagnosis (classifies patients into one of two categories):

**Brain Tumors**: Further divided into **benign (low grade)** and **malignant (high grade)**.

**Tumor-like lesions**: This category includes other brain abnormalities not classified as tumors.

The dataset is categorized into subfolders based on the two major classifications:

**Brain Tumors**: Containing both benign and malignant tumor.

**Benign (low grade).**

**Malignant (high grade).**

**Tumor like lesions**: This includes patient diagnosed with other brain conditions.

This dataset consists of one excel file and 55 folders. The excel files include patient's metadata for all patients such as patient's age, gender, and diagnosis while the 55 folders consist of four files, three for MRI scan (T2-FLAIR, pre- and post-contrast T1-weighted) and one for MRS scans. This structure allows for easy access and identification of specific cases.

## 4 Experimental Design, Materials and Methods

The dataset was collected at Al-Andalus Oncology Centre in Baghdad, Iraq, between 2023 and 2024. It included 55 patients diagnosed with either brain Tumors or tumor-like lesions. The data include both MRI (FLAIR, T1, T1 with contrast) and MRS modalities. The data provide a comprehensive imaging and metabolic information for researcher.

### 4.1 MRI acquisition

Data were collected from MRI FLAIR, and pre-and post-contrast T1 sequences. This modality is preferred because it suppresses fluid signals, which means high-contrast images with bright tumor borders, i.e., contrast enhancement. MRIs provide specific views of anatomy and are essential for determining the size, shape, and location of tumors and other findings.

### 4.2 MRS acquisition

MRS was performed on the same patients to capture the biochemical composition of the brain tissues. This non-invasive technique was employed to measure the levels of various metabolites within the brain lesions and surrounding tissues, enabling a deeper understanding of tissue metabolism. The MRS data complement the conventional MRI by offering information that aids in differentiating tumors and tumor like lesions based on their metabolic signatures.

### 4.3 Patient metadata

Along with the imaging data, metadata were collected for each patient, including:

• ID
• Age
• Gender
• Expert diagnosis, classifying the condition into brain tumors (benign or malignant) or tumor-like brain lesions.

The diagnosis process done by an expert with ten years of expectances in brain tumor diagnosis. To detect and classify a patient with a suspicious brain lesion, experts used both MRI and MRS to evaluate brain lesion characteristics, helping to identify whether a lesion is a brain tumor (benign or malignant) or a tumor-like lesion. MRI provides detailed anatomical images, with sequences such as T2-FLAIR pre- and post-contrast T1 which help in highlighting lesion boundaries and vascularity. Malignant tumors often show irregular shapes, strong contrast enhancement, and higher vascularity. In contrast, benign tumors show more regular shape, enhance less intensely. MRS offers metabolic data, measuring levels of key metabolites such as choline, creatine, N-acetylaspartate, and lactate. High choline with low NAA, for example, suggests aggressive tumor growth, while elevated lactate indicates anaerobic metabolism, commonly seen in high-grade tumors, infection.

### 4.4 Inclusion criteria

Patients were selected based on confirmed diagnoses of brain tumors or other brain abnormalities. The dataset includes benign (low-grade) and malignant (high-grade) tumors and other tumor-like conditions affecting the brain.

### 4.5 Data organization

The dataset is organized into subfolders for each patient, containing MRI and MRS data in a NIfTI file. The metadata file for all patients' ages, genders, and expert diagnoses is available in the main directory as an excel file.

This comprehensive data collection approach ensures that structural and metabolic information is available for each patient, offering a rich dataset for research in medical imaging, tumor classification, and diagnostic techniques.

## Limitations

This dataset consists of a limited number of images due to the rarity of the MRS device in Iraq that cause a difficulty in obtaining images. Moreover, the data were collected from a single geographic location (Baghdad, Iraq), which could introduce biases specific to regional medical practices or patient demographics. However, it can be consider a helpful resource for future research in the field.

## Ethics Statement

The authors confirm that all procedures involving human participants were performed by the ethical standards of the institutional and national research committees. Informed consent was obtained from all patients prior to data collection. The dataset was anonymized to protect patient privacy, and no identifiable information was included in the publicly available dataset based on the ethics committee of Al-Andalus Oncology Centre, Baghdad, Iraq, granted ethical approval for the study. It was granted on 4-April-2023.

## Credit Author Statement

Sura: Data collection, writing- reviewing and editing

Suhad: Reviewing and editing

Israa: Image diagnosis, reviewing and editing

## Data Availability

Mendeley DataBrain lesion MRI and co-related MRS Spectroscopy Dataset (Original data).
